# Growth-related quantitative trait loci in domestic and wild rainbow trout (*Oncorhynchus mykiss*)

**DOI:** 10.1186/1471-2156-11-63

**Published:** 2010-07-07

**Authors:** Brendan F Wringe, Robert H Devlin, Moira M Ferguson, Hooman K Moghadam, Dionne Sakhrani, Roy G Danzmann

**Affiliations:** 1 Department of Integrative Biology, 50 Stone Road East, University of Guelph, Guelph, ON, N1G 2W1 Canada; 2 Department of Biology, Memorial University of Newfoundland, St. John's, NL, A1B 3X9 Canada; 3 Fisheries and Oceans Canada, 4160 Marine Drive, West Vancouver, BC, V7V 1N6 Canada; 4 College of Veterinary Medicine, Cornell University, Ithaca, New York, USA

## Abstract

**Background:**

Somatic growth is a complex process that involves the action and interaction of genes and environment. A number of quantitative trait loci (QTL) previously identified for body weight and condition factor in rainbow trout (*Oncorhynchus mykiss*), and two other salmonid species, were used to further investigate the genetic architecture of growth-influencing genes in this species. Relationships among previously mapped candidate genes for growth and their co-localization to identified QTL regions are reported. Furthermore, using a comparative genomic analysis of syntenic rainbow trout linkage group clusters to their homologous regions within model teleost species such as zebrafish, stickleback and medaka, inferences were made regarding additional possible candidate genes underlying identified QTL regions.

**Results:**

Body weight (BW) QTL were detected on the majority of rainbow trout linkage groups across 10 parents from 3 strains. However, only 10 linkage groups (i.e., RT-3, -6, -8, -9, -10, -12, -13, -22, -24, -27) possessed QTL regions with chromosome-wide or genome-wide effects across multiple parents. Fewer QTL for condition factor (K) were identified and only six instances of co-localization across families were detected (i.e. RT-9, -15, -16, -23, -27, -31 and RT-2/9 homeologs). Of note, both BW and K QTL co-localize on RT-9 and RT-27. The incidence of epistatic interaction across genomic regions within different female backgrounds was also examined, and although evidence for interaction effects within certain QTL regions were evident, these interactions were few in number and statistically weak. Of interest, however, was the fact that these predominantly occurred within K QTL regions. Currently mapped growth candidate genes are largely congruent with the identified QTL regions. More QTL were detected in male, compared to female parents, with the greatest number evident in an F_1 _male parent derived from an intercross between domesticated and wild strain of rainbow trout which differed strongly in growth rate.

**Conclusions:**

Strain background influences the degree to which QTL effects are evident for growth-related genes. The process of domestication (which primarily selects faster growing fish) may largely reduce the genetic influences on growth-specific phenotypic variation. Although heritabilities have been reported to be relatively high for both BW and K growth traits, the genetic architecture of K phenotypic variation appears less defined (i.e., fewer major contributing QTL regions were identified compared with BW QTL regions).

## Background

Growth, in salmonids and other animals, is a complex physiological process controlled by interdependent gene expressions interacting with environmental factors [1]. Major environmental factors that influence growth include qualitative and quantitative nutritional availability [2,3], seasonal changes [4], and intra- and inter-specific competition [5,6]. Despite the plethora of environmental factors that may directly or indirectly influence growth rates, quantitative genetics studies investigating the heritability of growth have revealed moderate to high levels of heritability [7,8]. Evidence gained from studies examining the associations between life-history variation (e.g., maturation timing) and growth differences in salmonids have demonstrated strong physiological couplings among these traits [9-12]. Growth and maturation timing is also coupled to behavioural mating tactics in salmonids in that precociously maturing fish have a tendency to sire a higher percentage of precociously maturing offspring, and these offspring often exhibit higher growth rates to maturation [13,14]. These findings have led to the tacit understanding that growth differentials are also under fairly strong genetic control.

Fish growth is usually characterized as a positive allometry of muscle in relation to organs and, unlike mammals and birds, fish exhibit indeterminate growth with the most rapid period of growth occurring in the early life-history stages [15,16]. In contrast to mammals, in which muscle growth involves hypertrophy of muscle fibres formed prior to birth, fish are capable of both hypertrophying existing muscle fibres and recruiting new muscle fibres (hyperplasia) throughout their lives [17,18]. Fish also exhibit compensatory growth, which can result in partial, full, or enhanced growth rates compared to control fish following recovery from partial or complete food deprivation [2]. Given that the physiological dynamics of growth in poikilothermic vertebrates may be exceedingly complex due to interactions with environmental influences, it is still encouraging to note that relatively strong heritabilities for body mass (or weight) index and condition factor have been observed [7,19], and that consistent strain-specific body shape conformations have been observed in salmonids even among varying growth rate trajectories within strains [20]. However, the low underlying genetic correlations reported among traits such as fillet weight, protein, ash, water, and visceral and abdominal fat with condition factor indicated that this generalized body trait was a poor predictor of specific body composition traits [7].

Several factors may explain why body conformations display such an apparent lack of underlying genetic control. First, it is known that several contributing physiological factors may alter fish condition factors throughout their lifetime. Notably, lipid stores may fluctuate within fish tissues both seasonally and as a direct reflection of their nutritional intake [21-25]. Seasonal changes associated with the age of the fish in relation to the onset of sexual maturation may also influence body condition. For example, muscle somatic growth may be greatly diminished at the onset of male maturation, with excess energy being expended upon gonadal maturation. These physiological changes are known to enhance male condition factors compared to those of conspecific females, especially in the year class of a cohort wherein a high percentage of males undergo early sexual maturation, compared to females [23]. Older fish may also possess higher tissue water contents than younger fish [7]. However, water content tends to be more a factor of nutritional status with water content generally negatively correlated to lipid content [7,21,26]. Also, growth in salmonids is also not linear, and may occur in pulses according to environmental cues such as lunar cycles and annual seasons [27-30], which may differentially cause slight increases in body length, at a given body mass. All of these factors may contribute to producing a fish growth dynamic that results in a changing condition factor index for the same individual temporally.

Coupled with information on the known physiological functions of key candidate genes that may influence growth in teleost fishes [31], it is possible to target the genetic mapping of these genes and investigate their associated distribution among linkage groups to identified quantitative trait locus (QTL) regions within or among a group of species. The mapping and co-localization of candidate genes involved in growth to identified QTL regions, and the co-localization of these regions to homologous linkage groups among salmonid species [32-36] have provided initial insights into the genomic architecture of growth-regulating regions within the salmonid genome. For example, both O'Malley et al. [33] and Drew et al. [36] have reported that a significant QTL for body weight is localized to linkage group RT-27. This linkage group contains one of the duplicated copies of IGF2 in rainbow trout [35], suggesting that the expression of this gene may account for the observed growth QTL effects associated with RT-27. Interestingly, this linkage group also possesses a moderate-effect QTL for maturation timing in rainbow trout [37]. Similarly, duplicate copies of growth hormone are localized to the homeologous linkage groups RT-2/9, and genetic markers close to these regions have been identified as body weight QTL regions in both rainbow trout and Arctic charr (*Salvelinus alpinus*) [34].

The current study examines the genetic architecture of growth in rainbow trout utilizing different commercial strains established in Ontario and British Columbia, Canada, as well as wild fish from Pennask Lake in British Columbia. The two half-sib family crosses from British Columbia represent the widest differences in growth rates among the various strains used given that the female parents for the two paternal half-sib families are derived from a wild strain which possesses very slow growth rates in comparison to the commercial strain. In addition, all the male parents of the 9 families examined in this study were inter-strain F_1 _hybrids. Also 4 of the 5 male parents were used to produce paternal half-sib families, which facilitates the examination of possible epistatic interactions across genomic regions within different female backgrounds. The findings highlight several body weight QTL regions in rainbow trout that were identified in multiple parents, and we compare and contrast these regions to identified homologous chromosome regions in other teleost fishes [38].

## Results

### Body Weight and Condition Factor QTL - Ontario and British Columbia Families

**Figure 1:**
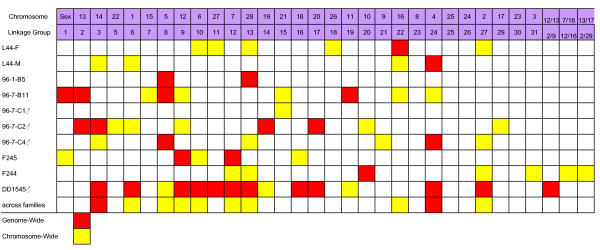
**Distribution of body weight (BW) QTL regions analyzed within 10 rainbow trout parental genomes, identifying genome-wide (red blocks), and chromosome-wide (yellow blocks) significant linkage group QTL regions within the experimental fish**. Information on the specific markers associated with each linkage group QTL region is provided in Additional File 1. QTL are assigned to both their linkage group [38,44] and chromosome size designations [39,40].

**Figure 2:**
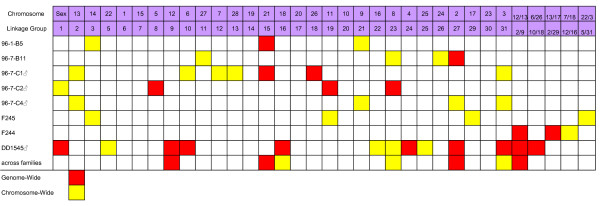
**Distribution of condition factor (K) QTL regions analyzed within 8 rainbow trout parental genomes, identifying genome-wide (red blocks), and chromosome-wide (yellow blocks) significant linkage group QTL regions within the experimental fish**. Information on the specific markers associated with each linkage group QTL region is provided in Additional File 2. QTL are assigned to both their linkage group [38,44] and chromosome size designations [39,40].

BW and K QTL were detected at the genome and chromosome wide level in the majority of RT LGs, and these regions are depicted in Figures 1 and 2, along with the current designations of these linkage groups according to the rainbow trout physical/cytogenetic map [39,40]. However, when considering growth across all measurement periods, only 10 linkage groups (i.e., RT-3, -6, -8, -9, -10, -12, -13, -22, -24, -27) possessed BW QTL that had either chromosome-wide or genome-wide significance in two or more parents. Similarly, only the K QTL localized to linkage groups to RT-9, -15, -16, -23, -27 and -31 were considered to be major QTL. Also, the localization of genome-wide significant K QTL to duplicated markers that are located on RT-2/9, along with chromosome-wide QTL in males 96-7-C1 and 96-7-C4 mapping to RT-2, suggests that this genomic region may also regulate body conformation dynamics. However, the precise localization of this effect is difficult to assess from this study, as the genome-wide effects were detected in the hybrid male DD1545 encompassing markers in the central region of RT-2/9. See Additional Files 1 and 2 for a description of the markers associated with each QTL region, their allelic substitution effects, and the proportion of experimental variance explained for each QTL. For the multiple growth stanzas surveyed, it should be noted that only those temporal periods with significant single point effects are reported.

A far greater number of BW QTL (total = 64 positions summed over all parents tested) compared to K QTL (total = 42) cumulative QTL position hits were detected among all parents, when considering both genome-wide and chromosome-wide significant regions. Also, as expected due to the lack of recombination in male salmonid genomes, the 5 males analyzed in this study revealed evidence for 63 QTL locations, while the 5 females tested produced evidence for only 43. This large difference, however, was primarily due to the inordinate number of significant QTL detected within the F_1 _hybrid male DD1545 from the British Columbia crosses.

### Body Weight QTL - O'Malley et al. (2003) Study

Reanalysis of the Lot44 data set [33] with newly added markers did not reveal substantial changes in the number of detected QTL. The only new QTL location to be identified was centered towards the telomeric end of linkage group RT-18. Markers OMM5009 and BX888425 on RT-18 were associated with a chromosome-wide QTL effect in the female parent. This region is, however, currently not recognized as a major QTL region, as only the female parent of Lot44 revealed evidence for a QTL at this location across the 10 parents tested.

### Epistasis

**Figure 3:**
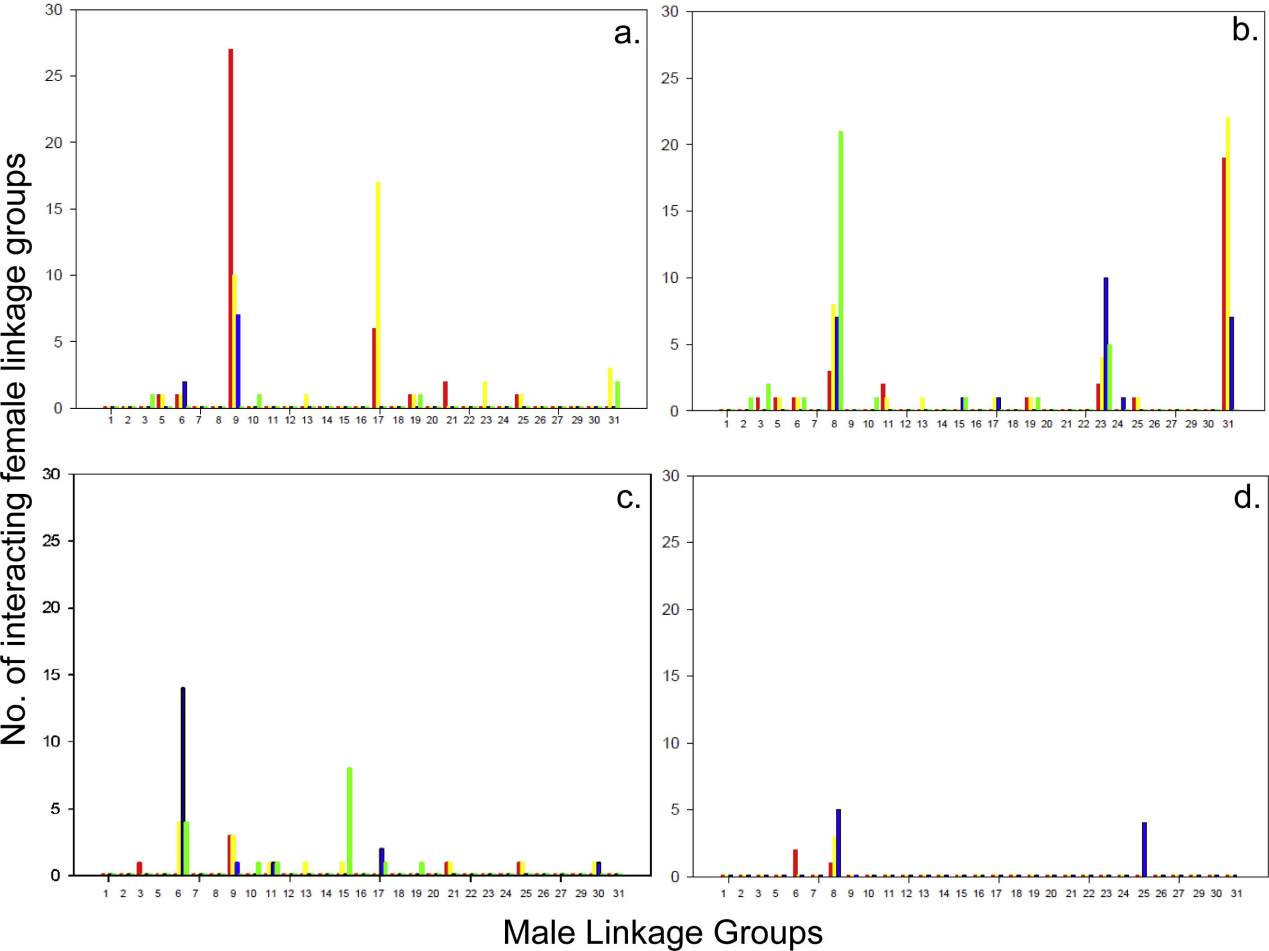
**Male-specific linkage groups associated with epistatic interactions in two different maternal backgrounds (P < 0.05) depicting the total number of marker-specific interactions evident for any given male linkage group**. Paternal half-sib interactions are shown for male DD1545 (a); 96-7-C1 (b); 96-7-C2 (c); and 96-7-C4 (d).

The evidence for epistatic interactions among pairwise locus comparisons across linkage groups within the male parents tested was relatively weak. Although there was evidence for a large number of interacting chromosomal regions with markers on linkage groups RT-9, and RT-17 in F_1 _hybrid DD1545 at the P < 0.05 level of significance (Figure 3a; Additional File 3), none of these interactions were significant at the P < 0.01 level of significance. Similarly, although there was evidence for a fair number of interactions among linkage groups with markers on RT-8, -23 and -31 in male 96-7-C1 (Figure 3b; Additional File 4), and interactions among linkage groups with markers on RT-6 and RT-15 in male 96-7-C2 (Figure 3c; Additional File 5), at the P < 0.05 level of significance, only marker interactions on RT-8, with RT-9, -16, and RT-23 with RT-16 remained significant at the P < 0.01 level of significance in 96-7-C1. Similarly, only interactions between markers on RT-15 with RT-19 loci and RT-6 with RT-8 remained significant at the P < 0.01 level in 96-7-C2. Male 96-7-C4 possessed only a few interaction effects with markers on RT-8 across two or more intervals (Figure 3d; Additional File 6), and none of these interactions remained significant at the P < 0.01 level. Also, none of the tests performed exceeded the genome-wide Bonferroni level of P~0.002. A summary of all the linkage groups showing moderate epistatic interactions across the various experimental data sets is given in Additional File 7.

## Discussion

### QTL influencing body weight (BW) and condition factor (K)

Body weight related QTL regions were more numerous than condition factor QTL regions among the rainbow trout parental genomes assessed in this study. This finding relates to the fact that rank orders of body weight distributions across sampling periods remained much more consistent among siblings within an experiment than did their overall condition factor distributions (data not shown). Although some fish displayed a uniform positive or negative condition factor profile throughout the experiment, others were more susceptible to exhibiting varying condition factor status dependent upon their growth phase. Kause et al. [7] have also reported that genetic correlations among different body composition traits and condition factor are relative low, suggesting condition factor itself may be a poor predictor of other body component traits (e.g., visceral fat, fillet weight, gutted weight, ash and water content, etc.), which may vary among siblings. The observation that certain individuals maintain either a temporally consistent positive or negative K distribution, while others exhibit fluctuating levels is of interest genetically, and will require further research.

Of the 6 linkage groups (i.e., RT-9, -15, -16, -23, -27 and -31) associated with stronger K influencing QTL regions, only two (i.e., RT-9 and -27) were also coupled to genes strongly influencing body weight. This is also supported by the fact that the genome-wide significant K QTL region detected with the singleton duplicated marker CA376300 in female F244 which is located on the homeologous chromosome arms RT-2q/9q, may also assigns to RT-9q. However, this assignment cannot exclude the region on RT-2q as having a similar influence on K distributions.

The localization of both BW and K QTL to linkage groups RT-9 and -27, has been supported by previous studies examining growth in both Arctic charr and Atlantic salmon. Both BW and K QTL have been detected on Atlantic salmon linkage groups AS-4/11, and AS-1 [41]. AS-1 shares homeology with AS-12, and both of these linkage groups are the orthologous linkage groups to the duplicated rainbow trout linkage group arms RT-27p and -31p. Similarly, Atlantic salmon chromosome arms AS-4q and AS-11q are the orthologous chromosome arms to rainbow trout RT-2q/9q homeologs [38]. Additionally, although not as well characterized, it is possible to relate the BW and K QTL detected on AC-20 in Arctic charr [34] to the homologous locations of this linkage group in rainbow trout and Atlantic salmon (i.e., the RT-2/9 and AS-4/11 linkage groups)(see Additional File 8). AC-20 appears to be a metacentric chromosome composed of fused homeologs in Arctic charr [42]. This would make both arms of AC-20 homologous to RT-2q/9q and AS-4q/11q. Similarly, a section of AC-4, was observed to possess both K and BW QTL in Arctic charr, and this linkage group shares homology to RT-27 (see Additional File 9). Interestingly, *IGF2 *has been mapped within this QTL region in Arctic charr and a copy of this gene also localizes close to the centromere on RT-27 in rainbow trout, although on the RT-27q arm [35]. The AS-2q arm shares homology with the RT-27q arm [38], and therefore, there may in fact be at least two regions influencing K in rainbow trout on RT-27, given that both the RT-27p/-31p chromosome arms appear to possess K QTL, as well as the region localized to the RT-27q arm.

Partial correspondence for associated growth traits were also evident in the comparative analyses wherein either an Atlantic salmon or Arctic charr linkage group possessed significant BW and K QTL influences that were partially supported with associated effects on either K or BW in the homologous rainbow trout linkage group regions. In the first case, a QTL for both BW and K detected on Atlantic salmon linkage group AS-8qa (markers Ssa401UoS, BHMS313A) appears to share homology to the short arm of rainbow trout linkage group RT-23 (i.e., RT-23p)[41]. Similarly, BW and K QTL have been localized to the duplicated homeologous linkage groups AS-22/24 [43]. These homeologs in Atlantic salmon share homology to the duplicated homeologs RT-7/15 [38]. While BW QTL have not been localized to these linkage groups in rainbow trout, significant genome-wide and chromosome-wide K QTL effects have been detected on RT-15 and RT-23, respectively.

Interestingly, the three strongest BW QTL regions detected in Arctic charr [34], (with explained trait variation ranging from 10.2 - 34.4%), correspond to BW QTL detected in this study. For example, the strongest BW QTL was detected on Arctic charr linkage group AC-8 (markers Omi26TUF, BHMS206 and Omi159TUF) shares homology to both rainbow trout linkage groups RT-3 and RT-17. While markers Omi26TUF and Omi159TUF are currently not located on the rainbow trout map, BHMS206 localizes to the central region of RT-3, suggesting related affinities to this region of RT-3. Indeed, the strong genome-wide significant BW QTL located on RT-3 spans the central portion of this linkage group (see Additional File 1). Additionally, the strong BW QTL detected on AC-13 (around marker OMM1211) localizes to a homologous region on the RT-24p arm. In the current study, a QTL region around this marker as well as on the RT-24q arm were detected (see Additional File 1) suggesting the possible existence of multiple loci influencing growth on this linkage group. Of note, is the fact that the strongest BW QTL detected in this study were also located on RT-3 and -24 (i.e., possessing genome-wide significant effects across families). Finally, although the apparent BW QTL effects were not as pronounced in rainbow trout, there is also evidence for a BW QTL region on RT-6 (homologous to the OmyRGT55TUF region in Arctic charr).

The strongest K QTL region that has been detected in Arctic charr [34] shares homology to the RT-5 linkage group. While only a moderate chromosome-wide QTL effect was detected on RT-5 in male DD1545 in this study, this chromosome appears to share extensive homeology to the RT-31q chromosome arm. RT-31 was one of the 6 linkage groups with detectable K QTL effects across families.

Four Arctic charr linkage groups (i.e., AC-4, -8, -20, and -36) were detected as having an influence on both BW and K QTL [34]. As previously mentioned, the region on RT-3 homologous to AC-8 does appear to have a strong influence on body weight, but there was only marginal evidence for K associated effects. Similarly, the region homologous to AC-36 (i.e., RT-5/31), has more pronounced effects on K rather than BW. However, the rainbow trout linkage group regions homologous to both AC-20, and possibly AC-4 (i.e., RT-9 and -27, respectively), were also observed to have a strong influence on both BW and K QTL locations in the current study. With respect to AC-4 homologies, it should be noted that this linkage group also shares syntenic blocks with regions of both RT-25 and RT-26.

### Candidate genes and growth

Genes with known growth regulating functions have been mapped to a majority of the rainbow trout linkage groups possessing quantitative trait locus regions for BW and K which is not surprising given the polygenic nature of these traits (see Additional Files 10 and 11). Many of the genes detailed in this table have been cited as being key candidate genes regulating vertebrate growth cycles, and thus likely to be important in regulating teleosts-specific growth rates [31]. For example we detected co-localization of genome wide significant BW QTL on LG RT-9, to which the candidate gene growth hormone (GH1) has been mapped [44,45], while the duplicated copy of the gene (GH2) localizes to another BW QTL region in rainbow trout on the linkage group (RT-2) that possesses homeology with RT-9 [45]. Also of note was the fact that copies of IGF1 and IGF2 have been mapped to linkage groups RT-15, and RT-27, respectively, in rainbow trout [35], and these genes along with GH are recognized as major regulators of the somatotrophic axis in fishes [31]. Thus, both the GH and IGF candidate genes appear to fall within QTL regions regulating both K and BW in rainbow trout, as well as other salmonids (see Additional Files 8 and 9).

The single strongest body weight QTL region detected in our study was located on RT-12 (see Additional File 1). A copy of the *Pax7 *gene has been mapped to this linkage group in rainbow trout [38]. Recent studies have documented the importance of *Pax7 *gene expression in maintained the integrity of mammalian myocyte satellite cells, as a stem cell reserve in tissue repair [46,47]. In contrast to mammals, where myocyte recruitment from mitotic reserves is very minor in mammalian muscle growth compared to hypertrophy, hyperplasia is a major mechanism of muscle growth along with hypertrophy for the majority of fish species that exhibit indeterminate growth [18], and is linked to *Pax7 *expression in teleosts [48]. This indicates that variation at the *Pax7 *gene may potentially be a very strong candidate for the major BW QTL differences observed among some of our rainbow trout families. Indeed, the synteny block containing *Pax7 *in zebrafish, medaka, and stickleback are homologous to the rainbow trout QTL region on RT-12 containing this gene (see Additional Files 12, 13, 14, 15, 16 and 17).

### Comparative Genomics of growth-related Candidate Genes

To ascertain if there may be additional candidate genes with potential growth-related influences within the QTL regions identified in this study, we compared gene homologies among genetic markers assigned to the rainbow trout genetic map, with their genomic locations in other model teleost species (e.g., medaka, zebrafish, and stickleback). This approach allowed us to identify additional potential candidate gene locations that may be syntenic within the identified rainbow trout QTL linkage group regions. Furthermore, if the homologous growth-related QTL regions in rainbow trout do not correspond to candidate gene locations in the model teleost species, then it is expected that counts for identified candidate genes would be randomly distributed among QTL and non-QTL regions in rainbow trout. In an initial comparison, we used the information on shared syntenies among rainbow trout linkage groups arms with their assigned affinities to the zebrafish and medaka genomes [38], and then extended this comparison to the stickleback genome using information on shared arm homologies among stickleback, medaka and zebrafish. A listing of the potential candidates to rainbow trout linkage group arms possessing the identified highly significant QTL assignments was obtained for zebrafish (Zv8), medaka (HdrR), and stickleback (BroadS1) gene builds available from the ENSEMBL http://www.ensembl.org database, with specific chromosome segment gene downloads from the BIOMART database within ENSEMBL (v54) (BLASTN search with 'Distant Homologies' default options using an e^-6 ^cutoff).

The search was restricted to rainbow trout linkage groups with evidence for multiple associations among the parents tested (i.e. RT-2q; -3p/q; -6p/q; -8p/q; -9p/q; -10p/q; -12p/q; -13; -15p/q; -16p/q; -23p/q; -24p/q; -27p/q, and -31p/q) where significant effects were detected across multiple families. For the majority of linkage groups corresponding to metacentric chromosomes, both the -p and -q arms of the linkage group were assessed given that significant genome-wide effects were only evident in the males, or in the case of RT-8 based polymorphisms, lack of recombination in the female parents made assignment to either arm problematic. The only exception to this was for linkage group RT-2 where the QTL appeared to localize to the -q arm.

Examination of Additional Files 12, 13 and 14, which highlight the putative syntenic blocks of the zebrafish, medaka, and stickleback genomes, respectively, homologous to the BW and K QTL linkage group regions in rainbow trout, indicates that approximately half of the gene locations described in these model species have putative homologous locations within rainbow trout linkage groups. To explain, and noting that some described gene locations are redundant in the current databases, there are 24354, 23021, and 24654 described gene accessions in the medaka, zebrafish, and stickleback databases, respectively, based upon assigned chromosome locations. The corresponding synteny block hits to the rainbow trout genome (excluding overlapping regions) identifies 14866 (61%), 13014 (56.5%), and 13223 (53.6%) putative gene homologies with medaka, zebrafish, and stickleback, respectively (see Additional Files 12, 13, and 14).

To assess whether the synteny hits with the rainbow trout genome overlap regions that contain key candidate genes influencing growth and energy homeostasis, we determined the chromosomal location of several growth regulating genes in the model species. Our criteria for selecting possible candidates was based upon knowledge of the direct physiological functions of key genes that are regarded as being important in regulating finfish growth and cell cycling [31,49], and possible candidates that are currently recognized as being of potential importance in regulating cellular energy states and metabolic flux [50]. It should also be noted that this list is far from complete, as the genes involved in intermediary metabolism, protein catabolic/anabolic processes, lipid transport, and many additional signal transduction, early embryogenesis and development categories were not addressed. Also, many of the genes involved in the sexual maturation cascade have been excluded, and as such these comparisons may be regarded as incomplete.

Approximately 100 major genes influencing growth and energy homeostasis (i.e., growth hormone complex, insulin growth factor complex, myogenic factors, etc.) were identified in the gene descriptions downloaded from BIOMART for the three model teleost species. The majority of these genes have putative homologies to syntenic rainbow trout QTL regions (i.e., approximately 60% within zebrafish, 72% in medaka, and 79% in stickleback), using the chromosomal locations of the genes that have been established in the latest builds from the 3 comparison teleost species (see Additional Files 15, 16, and 17), indicating that many important growth regulating genes fall within the putative rainbow trout QTL synteny blocks. While these associations appear not to be higher than by chance alone within the zebrafish genome hits, there are clearly a higher proportion of candidate gene homologies to the overlapping rainbow trout QTL blocks within the medaka (P < 0.05) and stickleback genome blocks (P < 0.001; χ^2 ^test; 1df).

### Epistasis

Epistatic interactions among pairwise male-specific marker comparisons across linkage groups were minimal and those interactions that were detectable were largely weak (i.e. only a few significant associations at P < 0.01 were observed, and none of the comparisons were considered significant after correcting for multiple comparisons across linkage groups). This suggests that the allelic influences of a genetic locus on a given phenotypic trait will largely be additive regardless of the parental background in which the marker is expressed. However, the observation that a small number of markers on specific linkage groups express reciprocal allelic associations with the magnitude of body weight across maternal backgrounds is intriguing. These effects were largely restricted to only a small number of linkage groups (2-4) within a given individual, and varied across temporal periods.

A far greater number of linkage group interactions were observed within male DD1545, compared to the three F_1 _hybrid brothers from family 96-7-C. The fact that male DD1545 was a hybrid between two source strains that differed substantially in intrinsic growth rates may contribute in explaining these findings. In other words, interaction effects may be less pronounced within an individual if the interacting alleles are more similar in their overall physiological influences. For commercial rainbow trout strains that have previously been selected for more uniform and rapid growth (i.e., the parents of family 96-7-C) this would be the expectation. These findings are also consistent with the observation that hybrid breakdown, through epistatic interactions, appear to be more pronounced the more distant genetically/geographically the hybridizing populations are that give rise to F_1 _individuals (summarized in [51]).

Most of the epistatic interactions in male DD1545 were localized to interactions with markers on linkage group RT-9. The fact that so many interactions were detected with RT-9, and to a lesser extent, RT-17 markers, cannot be directly addressed at present. Additional data is required from both intra- and inter-strain sources to assess the repeatability of the epistatic marker associations. The postulated and empirical basis for the origins of epistasis can relate to deleterious/detrimental gene combinations that can arise when the two strains are interbred following a prolonged temporal period of divergence [51,52]. The fact that multiple epistatic interactions were observed on RT-8 with two of the three siblings from family 96-7-C also suggests that familial and heritable differences may exist in the expression of epistasis. The finding that certain linkage groups may possess genes that are coupled to genome-wide interaction influences (i.e., RT-8, -9, -15, -17, -23, and RT-31) to a greater extent than other linkage groups will require further study in a greater number of half-sib family structures. Additionally, the observation that 4 of these 6 linkage groups (i.e., RT-9, -15, -23, and -31) were associated with body conformation differences is intriguing, and suggests that regions influencing condition factors may possess higher degrees of gene interaction effects in regulating the physiological expression of body shape.

### Inter-strain differences in the magnitude of QTL effects

Similar to the findings for epistasis, the greatest number of phenotypic QTL were detected in the interstrain hybrid male DD1545 produced from the mating between domesticated and wild rainbow trout parents. The reasons for this finding are likely three-fold. The first, and most likely contributing factor was the increased heterozygosity observed in the male compared to the two backcross Pennask Lake females. The increased levels of heterozygosity detected in the male would have increased the chances of detecting QTL. Secondly, given that male salmonids exhibit far lower rates of recombination than females [44], a given marker in the male is linked to a larger physical expanse of chromosome. Assuming candidate genes are equally dispersed throughout the genome, a given male maker would therefore represent more candidate genes that are in the same phase on average, than those detected with a female marker. Therefore, because less recombination would be associated with most male markers, QTL effects in males would be detected across greater physical distances of their chromosomes. Thirdly, and of most relevance biologically, is the likely contribution of past selection histories on growth differentials in the contributing parental strains to male DD1545. Pennask Lake fish being of a wild origin, will have experienced minimal selection towards maximizing growth performance, while fish from the contributing BC Spring Valley strain would have certainly experienced artificial selection for enhanced growth. Hence both contributing parental strains would largely carry a set of genes with opposing genetic architectures (i.e., normal wild slow growth performance with low stress tolerance and low predation risk due to wariness vs. higher growth performance with increased stress tolerance and behavioural boldness). This would likely have increased the chances of detecting QTL regions within the F_1 _hybrid male.

The discovery of relatively strong body weight QTL regions on the homeologous linkage groups RT-12/16 within the test families derived from British Columbia also highlights the need to test additional family origins for growth related QTL regions, as different underlying QTL regions may be observed in varying populations. Aside from the study by Drew et al. [36], which reported the existence of body weight QTL regions on the homeologous linkage groups RT-27/31 in a hybrid doubled haploid cross between two west coast rainbow trout strains (Arlee [AR] and Oregon State University line [OSU]), and the study by Nichols et al. [53] revealing body weight QTL on RT-8, and RT-20, and condition factor QTL region on RT-20 in an OSU × CW (Clearwater) rainbow trout experimental family, there have been few studies directed at the genomic architecture of growth-related QTL in rainbow trout. Nonetheless, these few studies do highlight that inter-strain differences may exist.

While the 3 groups of inter-strain crosses examined in the current study demonstrate the existence of body weight QTL on all the linkage groups reported by Drew et al. [36] and Nichols et al. [53], our findings differ from those exemplified in these two studies, in that only the QTL on RT-8 and RT-27 appear to have repeatable effects across a greater number of test parents (i.e., chromosome-wide effects across multiple families). This may in part be due to the fact that only single families were examined in these latter studies, and thus examination of a greater number of genomes may reveal the presence of additional QTL locations in these strains. It is also possible that the genomic architecture of all the doubled haploid lines used in these studies was very similar, as suggested by [36], which would limit the detection of specific growth-related QTL differences in these crosses. Presently, comparisons among multiple family origins across strains may be difficult to assess with respect to the information that they portend regarding QTL regions. With regard to the findings from the present study, however, it may be prudent to regard major QTL regions, as those that are detected in 30-40% of the individuals/families sampled. This value is based upon the fact that linkage groups which expressed BW QTL in 3 or 4 parents, were also observed to exhibit significant QTL effects across all families tested.

## Conclusions

An examination of the genomic regions associated with differences in body weight and condition factor among several families of rainbow trout has revealed that substantial genetic variation underlies the expression of these two phenotypic traits. Body Weight QTL regions (10 linkage groups) appear more numerous than condition factor QTL regions (6 linkage groups), with two linkage groups (i.e., RT-9 and -27) possessing QTL for both traits. These findings relate to the fact that the rankings of condition factors within individual fish are more variable as they grow compared with their weight rankings. Furthermore, these effects appear largely additive as only weak epistasis was detected for body weight QTL regions. Comparative synteny analyses of the rainbow trout QTL regions to their putative homologous chromosomal segments in zebrafish, medaka, and sticklebacks, indicates that a significantly greater proportion of approximately 100 *a priori *selected candidate genes (influencing metabolism and growth) within medaka and three-spine stickleback were homologous to rainbow trout QTL vs. non-QTL regions. A similar finding was not evident for zebrafish.

## Methods

### Experimental Families

Fish derived from two different regions in Canada (i.e., British Columbia and Ontario) were utilized in this study. The families produced in British Columbia were expected to be the most diverse genetically with respect to genes regulating growth as they involved a hybrid cross between a commercial fast growing strain and slower growing wild parents. The Ontario crosses involved progeny derived from inter-strain crosses of two commercial lines maintained at separate fish hatcheries within the province. The progeny were G_2 _offspring of line crosses originally established in the 1993/94 (= G_0 _generation) spawning season. Detailed descriptions of the experimental lines and sampling regimes for the growth analysis are provided below.

### British Columbia families

Two half-sib backcross families of rainbow trout were produced on 9 June, 2005. Both families were sired by a hybrid male (DD1545, Pennask Lake [wild] × Spring Valley Trout Farm, Langley, British Columbia [domestic]) backcrossed to two wild, Pennask Lake (south-central interior, British Columbia, Canada) dams. The two half-sib families created were designated PSV-2 and PSV-3 (Pennask Lake BC × Spring Valley BC = PSV).

These fish were raised at The Department of Fisheries and Oceans Center for Aquaculture and Environmental Research (DFO CAER) in West Vancouver, British Columbia. Each half-sib family was reared in a separate tank. The fish were hand fed daily to satiation with a commercial diet (Skretting Vancouver British Columbia) and raised under natural photoperiod.

On May 4^th ^and 5^th ^2006 adipose fin samples were taken from the fish in families PSV-2 and PSV-3, respectively, and the fish were implanted with passive integrative transponder tags (PIT tags). At the time of sampling, the PSV-2 fish averaged 12.0 g in weight and 10.0 cm in length, while the fish in PSV-3 averaged 9.5 g and 9.8 cm. One-hundred-forty-four fish in each family were randomly selected to be included in the analyses. Subsequent phenotypic measurements of growth (body mass to the nearest 0.1 g, and fork length to the nearest mm) were made on August 9/10, 2006, and February 13/14, 2007. A final measurement (body mass to nearest g, and fork length to nearest 0.1 cm) was made August 8/9, 2007. These four weighing periods are designated as W1, W2, W3, and W4, respectively, in some of the Additional File tables.

### Ontario families

Six half-sib diallel families were created on October 7, 1999 from individuals of known third generation pedigree derived from two pure commercial strains. The commercial strains utilized were Spring Valley (currently Lyndon strain maintained at the Lyndon Fish Hatcheries Ltd., New Dundee, Ontario) and Rainbow Springs (derived from Rainbow Springs Trout Farm, Thamesford, Ontario). It should be noted that to our knowledge, we do not believe that the British Columbia strain designated as Spring Valley is related via pedigree to the Ontario Spring Valley strain. The six half-sib families were produced by mating a pure strain Spring Valley female (96-1-B5), and an inter-strain F_1 _hybrid female (96-7-B11) to three different F_1 _inter-strain hybrid males (97-7-C1, 96-7-C2, and 96-7-C4), all derived from the same family (i.e., family 96-7-C). These families were initially produced to examine maturation timing QTL in rainbow trout, and further details on the study may be found in Haidle et al. [37]. (see Table one in [37] for a description of the family crosses).

Fish were reared at the Alma Aquaculture Research Station (Alma, Ontario). Fish were fed a ration corresponding to the thermal growth coefficients devised for rainbow trout (approximately 2-3% of body weight daily) [54], and while the six families were initially reared separately, all were raised at similar densities in the wet lab facilities, under a natural photoperiod regime. Feeding was adjusted bimonthly according to the mean biomass of fish per tank. Rations were reduced as the fish grew and reached maturation.

On June 20, 2000, the fish were weighed, PIT tagged, and twenty-five of the largest and twenty-five of the smallest fish from a pool of approximately 100 fish for each family were selected and retained for the remainder of the study. This tail-end sampling regime may be expected to increase the number and magnitude of the QTL effects detected, but given the fact that multiple families were being analyzed, repeatability on the location of the QTL effect was considered the most reliable indicator for the actual QTL effect (see below), and is also a check on false positive identification (i.e., repeated similar QTL regions are unlikely to arise by chance across families). On October 13, 2000, 50 fish from each half-sib family were randomly assigned to one of two single 2 m diameter tanks at the Alma hatchery, such that half the progeny from each family was placed into one tank. Thus each tank housed 150 fish for the final growth measurements. At the time of fish assignments to each replicate tank, all the fish were weighed (to the nearest g) and measured (fork length to the nearest 0.1 cm). Three subsequent growth measures were made on March 13, 2001, June 6, 2001, and October 11, 2001, followed by two additional measurements in 2002. However, the 2002 measurements were excluded from the analysis, as a high percentage of fish at this age (i.e., 2 + years old) displayed differential levels of sexual maturation. Additionally, the October 11, 2001 sample for families 99-4 and 99-8 (sired by male 96-7-C4) were excluded from the analysis as significant weight differences were detected between male and female offspring within these two half-sib families and this time point. These four weighing stanza are designated as W1, W2, W3, and W4, respectively, in some of the Additional File tables. In total, 287 individuals out of an initial 300 were used in the final analysis. Mean body weights (±SD) of all the experimental fish within each family lot at each weighing stanza are given in Additional File 18.

### Re-assessment of growth QTL from the O'Malley et al. (2003) study

One of the first studies reporting the location of body weight (BW) QTL in rainbow trout was published by O'Malley et al. [33]. These researchers reported the location of 7 BW QTL in the species, based upon an assessment of female body mass (N = 45) at two years of age within a single experimental family of rainbow trout (Lot44). Males were excluded from the analysis as many of these individuals had already shown pronounced signs of precocious maturation at the time of the experimental weighings. We have updated the data from the O'Malley et al. [33] study using information from approximately 4× the number of markers (i.e., ~1000) as those reported in the original study, as Lot44 is one of the two primary rainbow trout mapping panels used for comparative genomic studies in salmonid fishes at the University of Guelph [38]. The inclusion of this data gives a broader comparative family base to identify QTL locations for growth in rainbow trout.

### Calculation of Condition Factor

The condition factor (K) of each fish was obtained by determining the Z-standardized residual of the fish from the regression of Log_10 _transformed weight (g) of the fish (dependent variable) on the Log_10 _transformed length (cm) of the fish (independent variable) [55]. Separate regression equations were calculated for each family at each weighing time point. The standardized residuals convey the body shape status of the fish, as all positive residuals indicate fish which are more 'plump' than the average for their family, while negative residuals denote fish that are 'slimmer' than the average for their family. In salmonids, and all fish with a generalized fusiforme body shape, the slope of this regression is expected to be close to 3.0.

### Genomic Techniques

DNA was extracted using a standard phenol/chloroform protocol [56]. Polymerase chain reactions (PCR) reactions were carried out in 7 uL reactions with either one of the primer pairs being 5'-fluorescently end-labeled with either tetrachloro-6-carboxy-fluorescein (TET) or 6-carboxy-fluorescein (FAM). Each PCR reaction mixture consisted of 18 ng genomic DNA, 1.1× PCR buffer, 1.5-2 mM Mg, 1 mg/ml BSA, 0.125 mM dNTP, 0.17 μM forward and reverse primer, and 0.02 units of Taq. In general the PCR conditions were: an initial 10 minute denaturing step at 95°C, followed by thirty cycles of denaturation at 95°C for 1 minute, annealing for 30 seconds at 50-60°C and elongation at 72°C for 30 seconds, followed by a final elongation step at 72°C for 5 minutes. PCR conditions were standard throughout the study for all markers with only minor adjustments in annealing temperature and number of PCR cycles (i.e., 35 PCR cycles were used for markers MyoD1, and CNE268-274). Details on the primer sequences used their source references are available in the Appendix tables found in two folders, (Haidle-et-al_(2008).. and Wringe-Thesis) at [57]. Additional details on the primer sets used for the analysis of the Lot44 mapping panel can be found in [38], and details on the separation and visualization of the PCR products can be found in [34].

### Linkage Analysis

Compiled genotypic data were analyzed using the Visual Basic program, LINKMFEX [58], and sex-specific linkage maps were generated because of the large differences in recombination rate between the sexes [44]. Pairwise locus comparisons with a logarithm of odds ratio (LOD) score ≥3 was accepted as demonstrating linkage. The choice of markers to include in the study was based upon their known assignments to the rainbow trout linkage maps generated with mapping panels Lot25 and Lot44 [38], and the assignment of linkage groups to the physical map in rainbow trout [40]. For smaller acrocentric based chromosomes (e.g., RT-18, RT-26) a single genetic marker was targeted, while for larger metacentric chromosomes (e.g., RT-6, -8, -9) a minimum of 2-3 genetic markers were targeted, such that a central marker within each chromosome arm was genotyped. For the Ontario strains a total of 96 different microsatellite markers were genotyped across all parents, while for the British Columbia half-sib families, 137 different microsatellite markers were assessed for genetic variability across the 3 different test parents.

### QTL Analysis

Associations between trait variation and allelic segregation at marker loci were analyzed in two-tiered fashion. First, an exploratory method, single-point estimates for putative QTL were assessed using the distribution free non-parametric Wilcoxon Rank Sums Test. Highly significant QTL locations detected with this method (P < 0.001 and P < 0.01), were corroborated in the context of a more formal interval analysis methodology using the software program MultiQTL ver. 2.5 [59], specifying a single QTL per linkage group test model. QTL effects across multiple environmental measurements were assessed using the multiple environment covariance trait analysis function in MultiQTL. In the case of BW measurements, the trait variables were first Z-standardized across environmental measurements. However, given that K trait values were *apriori *Z-standardized measures, these variables were analyzed untransformed. Only BW measurements were considered for the size analysis, rather than both BW and length, given the fact that these two traits generally have genetic correlations >0.98 [55]. Therefore, it would be expected that QTL positions localized for BW would give essentially identical locations for length, and this was confirmed by comparing of single-point QTL positions for both traits [data not shown]. Conversely, genetic correlations generally span zero for associations between either body weight or length with K [55], indicating the genetic independence of the K trait with either BW or length.

Marker restoration was chosen as a default in the analysis using the phase generated independent linkage maps produced by GENOVECT-batch in LINKMFEX. LOD thresholds of significance for each linkage group were determined using 1000 permutation replicates, and those passing a threshold of significance of P <= 0.05, were further tested for genome-wide significance. Genome-wide significance for the QTL detected for each trait was assessed using the B-H False Discovery Rate (FDR) test [60], as implemented in MultiQTL with the FDR-alpha set at 0.05. Linkage groups with marker intervals passing the FDR-alpha level are described as possessing genome-wide significance, while linkage groups possessing significant QTL based upon the permutation testing, yet failing to pass the FDR test are designated as possessing chromosome-wide significance.

### Determination of significant QTL regions within rainbow trout

The localization of significant QTL locations among all the families tested was assessed within the framework of permutation testing using the combined family analysis within MultiQTL [59]. This option currently does not allow the incorporation of multiple trait measurements within each family, and therefore, Z-standardized trait observations (obtained within each family at each temporal measurement point) were averaged for each fish prior to analysis. Furthermore, given the fact that different markers were analyzed among parents within each linkage group (dependent upon variable polymorphisms among the parents), it was not possible to obtain a complementary set of markers across all parents for an interval analysis. Therefore, a marker or single point analysis option was chosen for the permutation tests using the best consensus map information across families. For most linkage groups, consensus map orders were established using the more complete marker information obtained from the lot44 parents. Chromosome-wide significant regions were identified at P = 0.05 level by permutation tests within each linage group among all families, with genome-wide effects similarly assessed at the 0.05 level using a FDR test [60].

### Analysis of Epistatic Interactions

To test for possible epistasis in the pairwise allelic associations of markers across different parental backgrounds, we utilized the physiological epistasis model of Cheverud and Routman [61] to examine paternal half-sib genomic interactions. Although maternal half-sib interactions could also have been examined, in the context of the present study, the matings performed facilitated 4 independent male evaluations, whereas only the two females from the Ontario strains were available for a similar assessment. The model of Cheverud and Routman [62] was modified to assess only the general expression of the common parents alleles against the genomic backgrounds of two different parents involved in the half-sib matings. In other words, epistasis is evaluated as the deviation from additive expectations for pairwise combinations of alleles at two interacting loci in the common parent (i.e., di-locus epistasis), when these alleles are expressed in genomic background of the two alternate parents in the half-sib matings. To assess only the effect of alternate genomic backgrounds on the interacting alleles, one alternate parental background is coded as allele state 1, and the second parental background is coded as allele state 2. This then facilitates the calculation of 8 of the 16 full interaction cell states outlined for di-locus epistatic interactions, and makes possible the estimation of epistatic and non-epistatic additive values for each cell. Epistatic interactions were only assessed for progeny body weights, and trait variables were Z-standardized prior to analysis. Overall genomic significance for the multiple tests performed was assessed using a modified Bonferroni correction set at: 0.05/LG = 0.0017, where LG = the number of linkage groups tested. The software used to calculate the epistatic values observed in 4 paternal half-sib sets examined in the current study is available at [58] and further details on the model used are given in [61].

## Authors' contributions

BFW and RGD wrote the manuscript and performed the bioinformatics and statistical analyses. BFW and HKM conducted the genotyping studies, while DS produced the Britich Columbia family crosses, and conducted the growth trials. RGD, MMF and RHD designed the study and all authors read and commented on the manuscript.

## Supplementary Material

Additional file 1 **Body weight QTL regions identified within 10 parental genomes in rainbow trout sorted by linkage group**. Click here for file

Additional file 2 **Condition factor QTL regions identified within 8 parental genomes in rainbow trout sorted by linkage group**. Click here for file

Additional file 3 **Marker regions showing epistasis in male DD1545**. Click here for file

Additional file 4 **Marker regions showing epistasis in male 96-7-C1**. Click here for file

Additional file 5 **Marker regions showing epistasis in male 96-7-C2**. Click here for file

Additional file 6 **Marker regions showing epistasis in male 96-7-C4**. Click here for file

Additional file 7 **Oxford grid depicting male-specific linkage groups possessing significant epistatic interaction effects with the female linkage groups indicated**. Click here for file

Additional file 8 **Homologous comparisons of body weight and condition factor QTL locations detected in rainbow trout linkage group 9 with Atlantic salmon and Arctic charr linkage groups**. Click here for file

Additional file 9 **Homologous comparisons of body weight and condition factor QTL locations detected in rainbow trout linkage group 27 with Atlantic salmon and Arctic charr linkage groups**. Click here for file

Additional file 10 **Possible growth candidate genes locations in the rainbow trout linkage map**. Click here for file

Additional file 11 **Regions of putative homology between rainbow trout linkage group QTL positions, and chromosomal locations in stickleback (Ga), medaka (Olat), and zebrafish (Dr)**. Click here for file

Additional file 12 **Putative homologies of rainbow trout BW and K QTL regions to the zebrafish genome (ENSEMBL Zv8) build depicting +/-2 Mb windows to the regions of putative encompassed homology**. Click here for file

Additional file 13 **Putative homologies of rainbow trout BW and K QTL regions to the medaka genome (ENSEMBL HdR) build depicting +/-2 Mb windows to the regions of putative encompassed homology**. Click here for file

Additional file 14 **Putative homologies of rainbow trout BW and K QTL regions to the stickleback genome (ENSEMBL Broad S1) build depicting +/-2 Mb windows to the regions of putative encompassed homology**. Click here for file

Additional file 15 **Putative homologies of rainbow trout QTL regions to key growth and energy homeostasis regulating genes in the zebrafish genome**. Click here for file

Additional file 16 **Putative homologies of rainbow trout QTL regions to key growth and energy homeostasis regulating genes in the medaka genome**. Click here for file

Additional file 17 **Putative homologies of rainbow trout QTL regions to key growth and energy homeostasis regulating genes in the stickleback genome**. Click here for file

Additional file 18 **Comparison of body weight (BW) trait values across experimental families**. Click here for file
