# Supplementary material for: Growth-related quantitative trait loci in domestic and wild rainbow trout (Oncorhynchus mykiss)
Source: BMC Genet. 2010 Jul 7;11:63. doi: 10.1186/1471-2156-11-63 (PMC2914766; doi:10.1186/1471-2156-11-63)
Supplement: Additional file 8 — Homologous comparisons of body weight and condition factor QTL locations detected in rainbow trout linkage group 9 with Atlantic salmon and Arctic charr linkage groups. [file 1471-2156-11-63-S8.PDF]

Additional file 8. Body weight and condition factor QTL locations detected in rainbow trout (RT) linkage group 9 and their homologous linkage group locations in Atlantic salmon (AS) and Arctic charr (AC) genomes. QTL blocks (+/- 20cM from interval effect locations) are shown in green within each linkage group, and homologous markers are depicted in red. Centromeres are indicated as black segments. A more complete listing of markers assigned to these linkage groups may be found in [38] and [42].

Note: Female linkage maps are shown, and therefore some of the homologous markers represent markers currently only mapped in males from the comparison species.

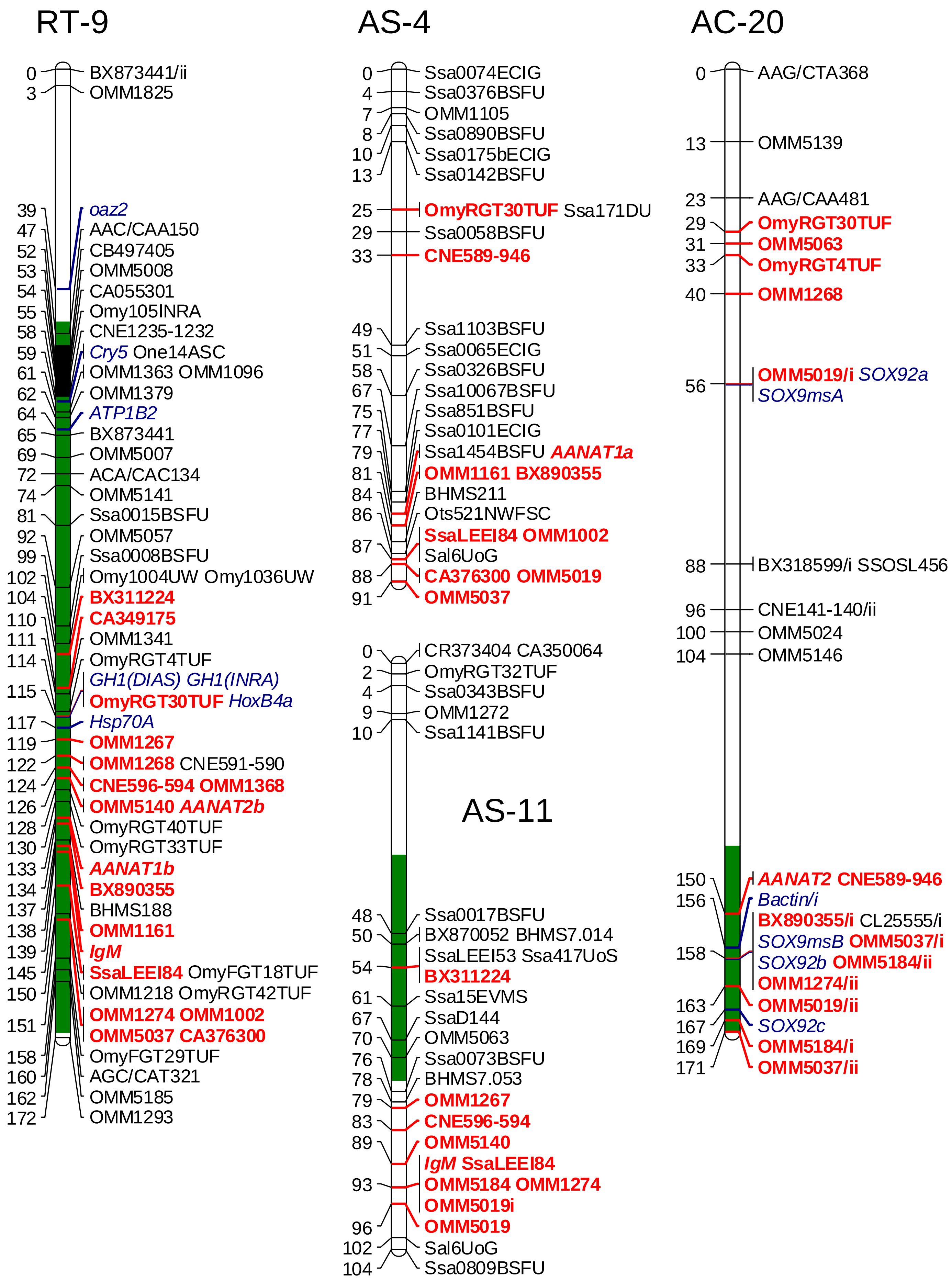

RT-9
0 BX873441/ii
3 OMM1825
39 oaz2
47 AAC/CAA150
52 CB497405
53 OMM5008
54 CA055301
55 Omy105INRA
58 CNE1235-1232
59 Cry5 One14ASC
61 OMM1363 OMM1096
62 OMM1379
64 ATP1B2
65 BX873441
69 OMM5007
72 ACA/CAC134
74 OMM5141
81 Ssa0015BSFU
92 OMM5057
99 Ssa0008BSFU
102 Omy1004UW Omy1036UW
104 BX311224
110 CA349175
111 OMM1341
114 OmyRGT4TUF
115 GH1(DIAS) GH1(INRA)
OmyRGT30TUF HoxB4a
117 Hsp70A
119 OMM1267
122 OMM1268 CNE591-590
124 CNE596-594 OMM1368
126 OMM5140 AANAT2b
128 OmyRGT40TUF
130 OmyRGT33TUF
133 AANAT1b
134 BX890355
137 BHMS188
138 OMM1161
139 IgM
145 SsaLEEI84 OmyFGT18TUF
150 OMM1218 OmyRGT42TUF
151 OMM1274 OMM1002
OMM5037 CA376300
158 OmyFGT29TUF
160 AGC/CAT321
162 OMM5185
172 OMM1293
AS-4
0 Ssa0074ECIG
4 Ssa0376BSFU
7 OMM1105
8 Ssa0890BSFU
10 Ssa0175bECIG
13 Ssa0142BSFU
25 OmyRGT30TUF Ssa171DU
29 Ssa0058BSFU
33 CNE589-946
49 Ssa1103BSFU
51 Ssa0065ECIG
58 Ssa0326BSFU
67 Ssa10067BSFU
75 Ssa851BSFU
77 Ssa0101ECIG
79 Ssa1454BSFU AANAT1a
81 OMM1161 BX890355
84 BHMS211
86 Ots521NWFSC
87 SsaLEEI84 OMM1002
Sal6UoG
88 CA376300 OMM5019
91 OMM5037
AS-11
0 CR373404 CA350064
2 OmyRGT32TUF
4 Ssa0343BSFU
9 OMM1272
10 Ssa1141BSFU
48 Ssa0017BSFU
50 BX870052 BHMS7.014
54 SsaLEEI53 Ssa417UoS
BX311224
61 Ssa15EVMS
67 SsaD144
70 OMM5063
76 Ssa0073BSFU
78 BHMS7.053
79 OMM1267
83 CNE596-594
89 OMM5140
IgM SsaLEEI84
93 OMM5184 OMM1274
OMM5019i
96 OMM5019
102 Sal6UoG
104 Ssa0809BSFU
AC-20
0 AAG/CTA368
13 OMM5139
23 AAG/CAA481
29 OmyRGT30TUF
31 OMM5063
33 OmyRGT4TUF
40 OMM1268
56 OMM5019/i SOX92a
SOX9msA
88 BX318599/i SSOSL456
96 CNE141-140/ii
100 OMM5024
104 OMM5146
150 AANAT2 CNE589-946
156 Bactin/i
BX890355/i CL25555/i
158 SOX9msB OMM5037/i
SOX92b OMM5184/ii
OMM1274/ii
163 OMM5019/ii
167 SOX92c
169 OMM5184/i
171 OMM5037/ii
