# Supplementary material for: Growth-related quantitative trait loci in domestic and wild rainbow trout (Oncorhynchus mykiss)
Source: BMC Genet. 2010 Jul 7;11:63. doi: 10.1186/1471-2156-11-63 (PMC2914766; doi:10.1186/1471-2156-11-63)
Supplement: Additional file 9 — Homologous comparisons of body weight and condition factor QTL locations detected in rainbow trout linkage group 27 with Atlantic salmon and Arctic charr linkage groups. [file 1471-2156-11-63-S9.PDF]

Additional file 9. Body weight and condition factor QTL locations detected in rainbow trout (RT) linkage group 27 and their homologous linkage group locations in Atlantic salmon (AS) and Arctic charr (AC) genomes. QTL blocks (+/- 20cM from interval effect locations) are shown in green within each linkage group, and homologous markers are depicted in red and purple for the p- and q-arms of RT-27. Centromeres are indicated as black segments. A more complete listing of markers assigned to these linkage groups may be found in [38] and [42]. Note: only the p-arm is depicted for AS-1.

Note: Female linkage maps are shown, and therefore some of the homologous markers represent markers currently only mapped in males from the comparison species.

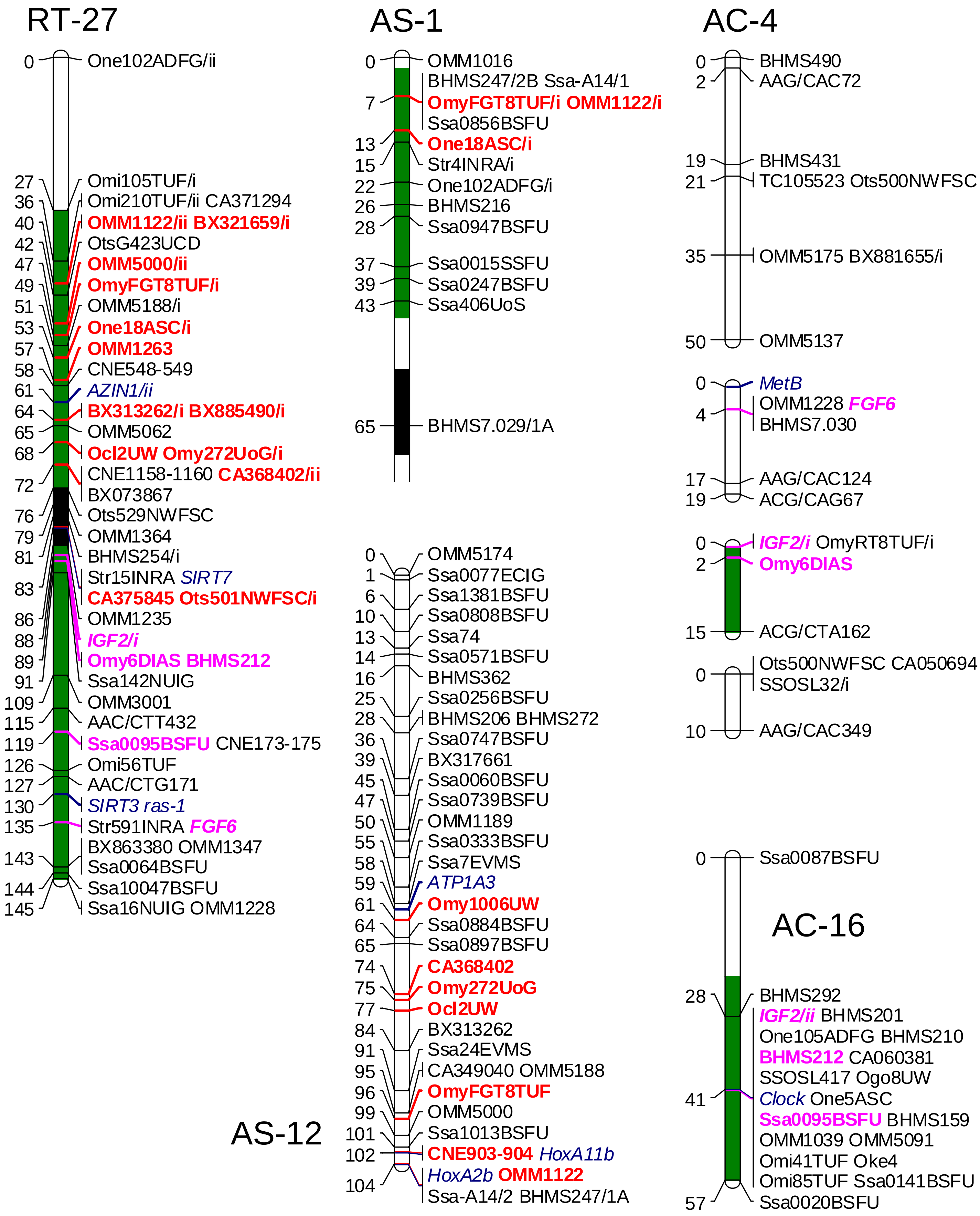
RT-27
0 One102ADFG/ii
27 Omi105TUF/i
36 Omi210TUF/ii CA371294
40 OMM1122/ii BX321659/i
42 OtsG423UCD
47 OMM5000/ii
49 OmyFGT8TUF/i
51 OMM5188/i
53 One18ASC/i
57 OMM1263
58 CNE548-549
61 AZIN1/ii
64 BX313262/i BX885490/i
65 OMM5062
68 Ocl2UW Omy272UoG/i
72 CNE1158-1160 CA368402/ii
BX073867
76 Ots529NWFSC
79 OMM1364
81 BHMS254/i
Str15INRA SIRT7
83 CA375845 Ots501NWFSC/i
86 OMM1235
88 IGF2/i
89 Omy6DIAS BHMS212
91 Ssa142NUIG
109 OMM3001
115 AAC/CTT432
119 Ssa0095BSFU CNE173-175
126 Omi56TUF
127 AAC/CTG171
130 SIRT3 ras-1
135 Str591INRA FGF6
BX863380 OMM1347
143 Ssa0064BSFU
144 Ssa10047BSFU
145 Ssa16NUIG OMM1228
AS-1
0 OMM1016
BHMS247/2B Ssa-A14/1
7 OmyFGT8TUF/i OMM1122/i
Ssa0856BSFU
13 One18ASC/i
15 Str4INRA/i
22 One102ADFG/i
26 BHMS216
28 Ssa0947BSFU
37 Ssa0015SSFU
39 Ssa0247BSFU
43 Ssa406UoS
65 BHMS7.029/1A
AS-12
0 OMM5174
1 Ssa0077ECIG
6 Ssa1381BSFU
10 Ssa0808BSFU
13 Ssa74
14 Ssa0571BSFU
16 BHMS362
25 Ssa0256BSFU
28 BHMS206 BHMS272
36 Ssa0747BSFU
39 BX317661
45 Ssa0060BSFU
47 Ssa0739BSFU
50 OMM1189
55 Ssa0333BSFU
58 Ssa7EVMS
59 ATP1A3
61 Omy1006UW
64 Ssa0884BSFU
65 Ssa0897BSFU
74 CA368402
75 Omy272UoG
77 Ocl2UW
84 BX313262
91 Ssa24EVMS
95 CA349040 OMM5188
96 OmyFGT8TUF
99 OMM5000
101 Ssa1013BSFU
102 CNE903-904 HoxA11b
104 HoxA2b OMM1122
Ssa-A14/2 BHMS247/1A
AC-4
0 BHMS490
2 AAG/CAC72
19 BHMS431
21 TC105523 Ots500NWFSC
35 OMM5175 BX881655/i
50 OMM5137
0 MetB
4 OMM1228 FGF6
BHMS7.030
17 AAG/CAC124
19 ACG/CAG67
0 IGF2/i OmyRT8TUF/i
2 Omy6DIAS
15 ACG/CTA162
0 Ots500NWFSC CA050694
SSOSL32/i
10 AAG/CAC349
AC-16
0 Ssa0087BSFU
28 BHMS292
IGF2/ii BHMS201
One105ADFG BHMS210
BHMS212 CA060381
SSOSL417 Ogo8UW
41 Clock One5ASC
Ssa0095BSFU BHMS159
OMM1039 OMM5091
Omi41TUF Oke4
Omi85TUF Ssa0141BSFU
57 Ssa0020BSFU
